# RNA Interference-Based Therapy for Spinocerebellar Ataxia Type 7 Retinal Degeneration

**DOI:** 10.1371/journal.pone.0095362

**Published:** 2014-04-23

**Authors:** Pavitra S. Ramachandran, Sajag Bhattarai, Pratibha Singh, Ryan L. Boudreau, Stewart Thompson, Albert R. LaSpada, Arlene V. Drack, Beverly L. Davidson

**Affiliations:** 1 Interdisciplinary program in Genetics, University of Iowa, Iowa City, Iowa, United States of America; 2 Department of Ophthalmology and Visual sciences, University of Iowa, Iowa City, Iowa, United States of America; 3 Department of Internal Medicine, University of Iowa, Iowa City, Iowa, United States of America; 4 Departments of Pediatrics, Cellular & Molecular Medicine, and Neurosciences, Division of Biological Sciences, the Institute for Genomic Medicine, and the Sanford Consortium for Regenerative Medicine, University of California San Diego, La Jolla, California, United States of America; 5 Department of Neurology, University of Iowa, Iowa City, Iowa, United States of America; 6 Department of Physiology and Biophysics, University of Iowa, Iowa City, Iowa, United States of America; National Eye Institute, United States of America

## Abstract

Spinocerebellar ataxia type 7 (SCA7) is an autosomal dominant neurodegenerative disease characterized by loss of motor coordination and retinal degeneration with no current therapies in the clinic. The causative mutation is an expanded CAG repeat in the ataxin-7 gene whose mutant protein product causes cerebellar and brainstem degeneration and retinal cone-rod dystrophy. Here, we reduced the expression of both mutant and wildtype ataxin-7 in the SCA7 mouse retina by RNA interference and evaluated retinal function 23 weeks post injection. We observed a preservation of normal retinal function and no adverse toxicity with ≥50% reduction of mutant and wildtype ataxin-7 alleles. These studies address an important safety concern regarding non-allele specific silencing of ataxin-7 for SCA7 retinal therapy.

## Introduction

Spinocerebellar ataxia type 7 (SCA7) is a neurodegenerative disease characterized by ataxia, which manifests as loss of motor coordination, dysarthria, slower reflexes, and retinal degeneration leading to vision loss. Anticipation is a key feature of this disease and for SCA7 patients with early onset disease, vision loss can occur early in life, followed by ataxia [1]. The retinal degeneration is a cone-rod dystrophy, which may manifest as a maculopathy when the onset is in adulthood, or as a diffuse retinopathy or geographic atrophy with childhood onset [2]. Typically a decrease in visual acuity is noted, followed by loss of peripheral vision and ultimately complete blindness in many cases [3].

SCA7 is caused by the expansion of CAG repeats in the ataxin-7 gene (*ATXN7*), which translates into a polyglutamine expanded protein. Studies in SCA7 mouse models and *in vitro* studies have demonstrated that polyglutamine expanded ataxin-7 disrupts the transcription of CRX, the cone-rod homeobox protein, in turn affecting the transcription of CRX-regulated genes resulting in a cone-rod dystrophy [4], [5].

There is no known treatment for SCA7 retinal degeneration. Previous studies have demonstrated that reduction of mutant ataxin-7 in the brain by Cre-Lox excision in a SCA7 mouse model can alleviate motor phenotypes [6]. However, from a clinical perspective, targeting the mutant allele alone will require targeting a patient specific polymorphism within the disease allele to discriminate it from wildtype. At the current time, the personalized development of RNA interference (RNAi) therapy on a per family basis is impractical. We therefore designed and tested an RNAi sequence that could reduce the expression of both mutant and wildtype ataxin-7 (non-allele specific silencing) and assessed feasibility *in vivo*.

The BAC-Prp-SCA7-92Q mouse model used in this work expresses the human mutant ataxin-7 cDNA in the central nervous system (CNS), including the retina, and displays ataxic phenotypes [7]. This model does not demonstrate retinal degeneration although the transgene is expressed in the retina as seen by RT-qPCR and western blot. Nonetheless, the model gives us a unique opportunity to test if reducing the wildtype and mutant ataxin-7 levels long-term in the SCA7 mouse retina is safe with maintenance of normal retinal function. We demonstrate for the first time that RNAi-based reduction of mutant and wildtype ataxin-7 expression is well tolerated in the SCA7 mouse retina and the introduction of an ataxin-7-targeted RNAi sequence does not affect normal retinal function.

## Results

### Silencing ataxin-7 expression in the SCA7 retina

We designed small interfering RNAs (siRNAs) targeting ataxin-7 using a low off-target prediction algorithm [8]. These siRNAs were cloned into artificial miRNA expression vectors [9] and were tested *in vitro*. We identified one sequence (miS4) that could reduce human mutant ATXN7 and mouse ataxin-7 mRNA expression by ∼50% *in vitro* relative to controls (Fig. S1a,b). To target the mouse photoreceptors, miS4 and a scrambled RNAi control sequence (miC) were cloned into adeno-associated viral (AAV) shuttle vectors and AAV2/1 viruses were generated expressing miS4 or miC. The reporter gene eGFP was expressed from a second cassette on the same vector for visualization of transduced cells.

**Figure 1:**
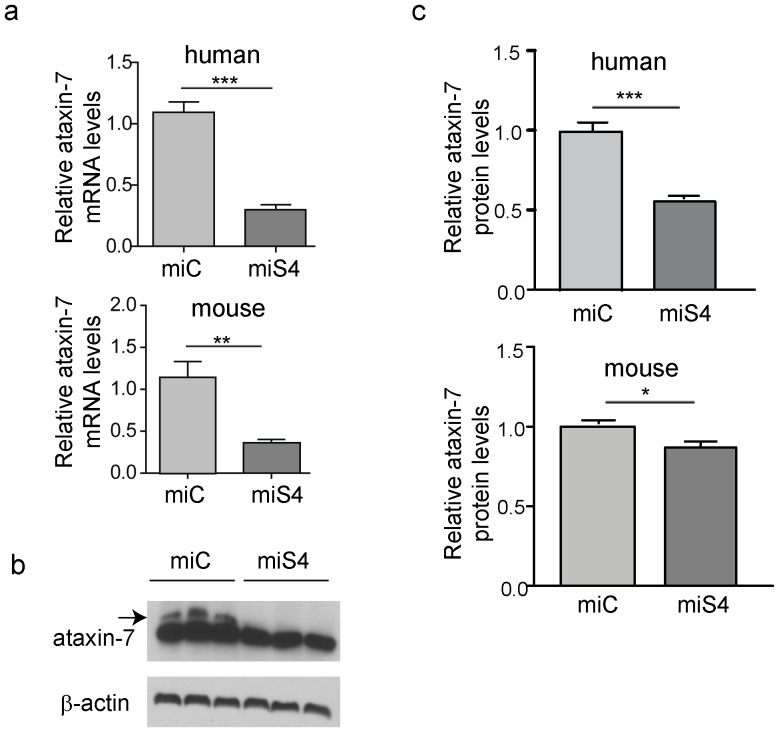
Reduction of ataxin-7 mRNA in the SCA7 mouse retina. (a) Relative levels of human (top panel) or mouse (bottom panel) ataxin-7 mRNA levels one month post-injection with AAV2/1 miC or miS4. (b) Representative western blot demonstrating mutant human (upper band, denoted by arrow) and mouse ataxin-7 (lower band) protein levels one month post injection of AAV2/1 miC or miS4. For quantification (c), ataxin-7 protein levels were normalized to β-actin (upper panel, human protein; lower panel, mouse protein). For all panels, results are represented as mean ±SEM (n = 3), ***p<0.001, **P<0.01,*p<0.05.

To target the photoreceptor cells in SCA7 mice (as SCA7 is a cone-rod dystrophy), AAV2/1.miS4 was subretinally injected into one eye and the contra-lateral eye was subretinally injected with AAV2/1.miC. One month post injection we observed a significant reduction of ataxin-7 mRNA (human mutant ∼60%, p<0.001; mouse ∼60%, p<0.01) and protein levels (human mutant ∼50%, p<0.001; mouse ∼15%, p<0.05) in the miS4 injected retinas relative to miC (Figs. 1a–c).

### Sustained silencing of ataxin-7 in the SCA7 retina

**Figure 2:**
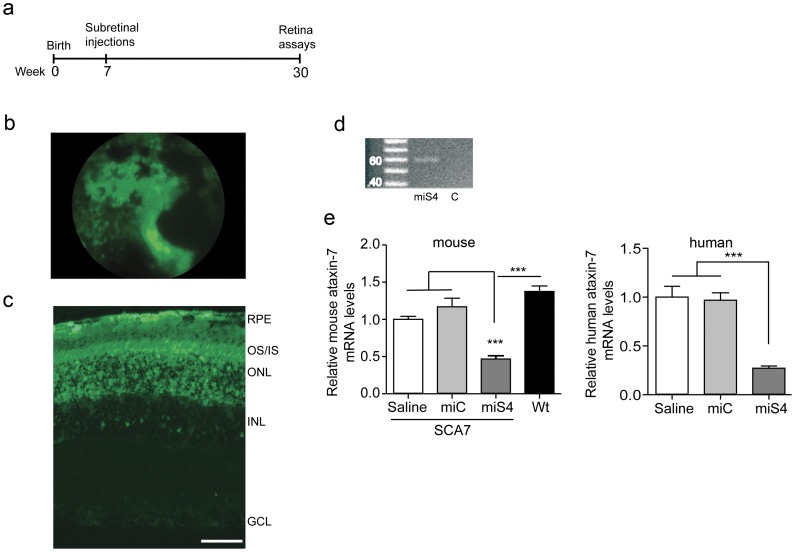
Sustained suppression of ataxin-7 expression. (a) Experimental scheme. (b) eGFP fluorescence as observed by funduscopy. (c) eGFP fluorescence indicating transduction of the RPE, photoreceptors, outer nuclear layer and few inner nuclear layer cells in the SCA7 retina at 30 weeks. (d) miS4 expression validated by stem-loop PCR in retinal extracts (n = 3). miC was used as the negative control. (e) Relative levels of mouse (left panel) or human (right panel) ataxin-7 mRNA levels. Results are represented as mean ±SEM (n = 3 per group), *** p<0.001.

To assess the long-term effects of non-allele specific silencing of ataxin-7 in the retina, we subretinally injected SCA7 mice at 7 weeks of age and followed them for 23 weeks (Fig. 2a). We included mice injected with subretinal saline injection as an additional control group in our long-term study. Funduscopy demonstrated effective transduction across a large area of the retina (Fig. 2b), and histological evaluation at 30 weeks of age showed extensive transduction of the retinal pigment epithelium (RPE), photoreceptor cells and cells within the inner nuclear layer (Fig. 2c). This contrasts results from prior reports of subretinal injections of AAV2/1, where the RPE was primarily transduced [10]. The differences could arise from differences in production of the AAV2/1 virus (we used the baculovirus system while Lebherz *et al*., used the triple transfection system) or differences in the method of the subretinal injection itself, as the viral genomes injected were similar between the two studies. The injected miS4 sequence was detected at 30 weeks by stem loop PCR in the retina (Fig. 2d), and RT-qPCR assays on harvested RNA revealed a significant reduction in ataxin-7 mouse (∼50%, p<0.001) and mutant human (∼65%, p<0.001) transcript levels in the miS4 injected retina relative to saline and miC injected groups (Fig. 2e).

### Reducing ataxin-7 expression does not induce neuropathology in the SCA7 mouse retina

**Figure 3:**
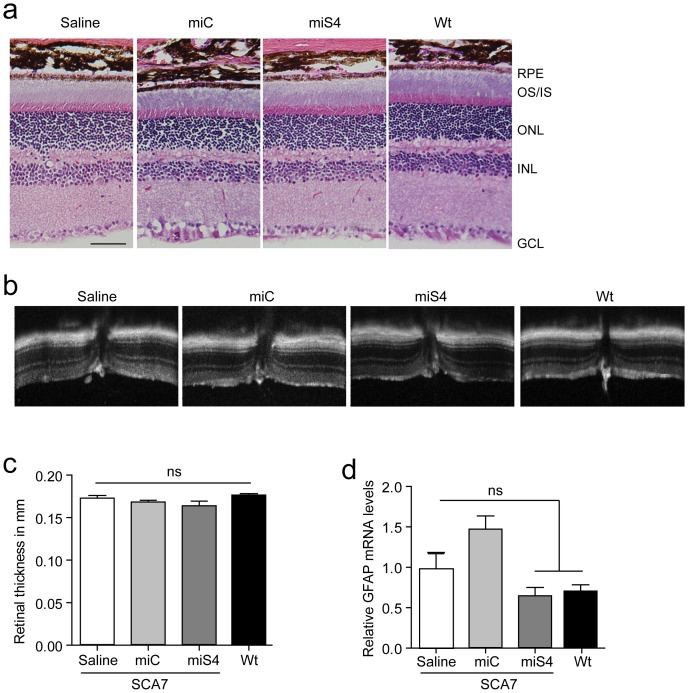
Assessing toxicity post-injection at 30 weeks. (a) Hematoxylin and eosin staining of retinas at 30 weeks (n = 3 per group) (b) Optical coherence tomography (OCT) images of the retinas at the region of the optic nerve (n≥6 per group). (c) Retinal thickness measured using the OCT images. (d) Relative GFAP mRNA levels in the injected retinas by RT-qPCR analysis. For (c; n≥6 per group) and (d; n = 3 per group), results are represented as mean ±SEM.

To assess if miS4 treatment induced toxicity in the retina, we examined retinal morphology by hematoxylin and eosin staining and observed no gross changes in treated vs. normal, untreated eyes (Fig. 3a). We also measured the retinal thickness by optical coherence tomography (OCT) at 30 weeks before euthanizing the mice. Retinal thickness was not different among the injected groups (Figs. 3b,c). To assess if gliosis was induced, we quantified the expression of the glial fibrillary acidic protein (GFAP) by RT-qPCR and observed no miS4-induced increase in GFAP expression (Fig. 3d) relative to saline injected eyes. Interestingly, eyes injected with the control miRNA expressing vectors miC did show elevated GFAP levels.

### Retinal function is not altered in miS4 treated retinas

**Figure 4:**
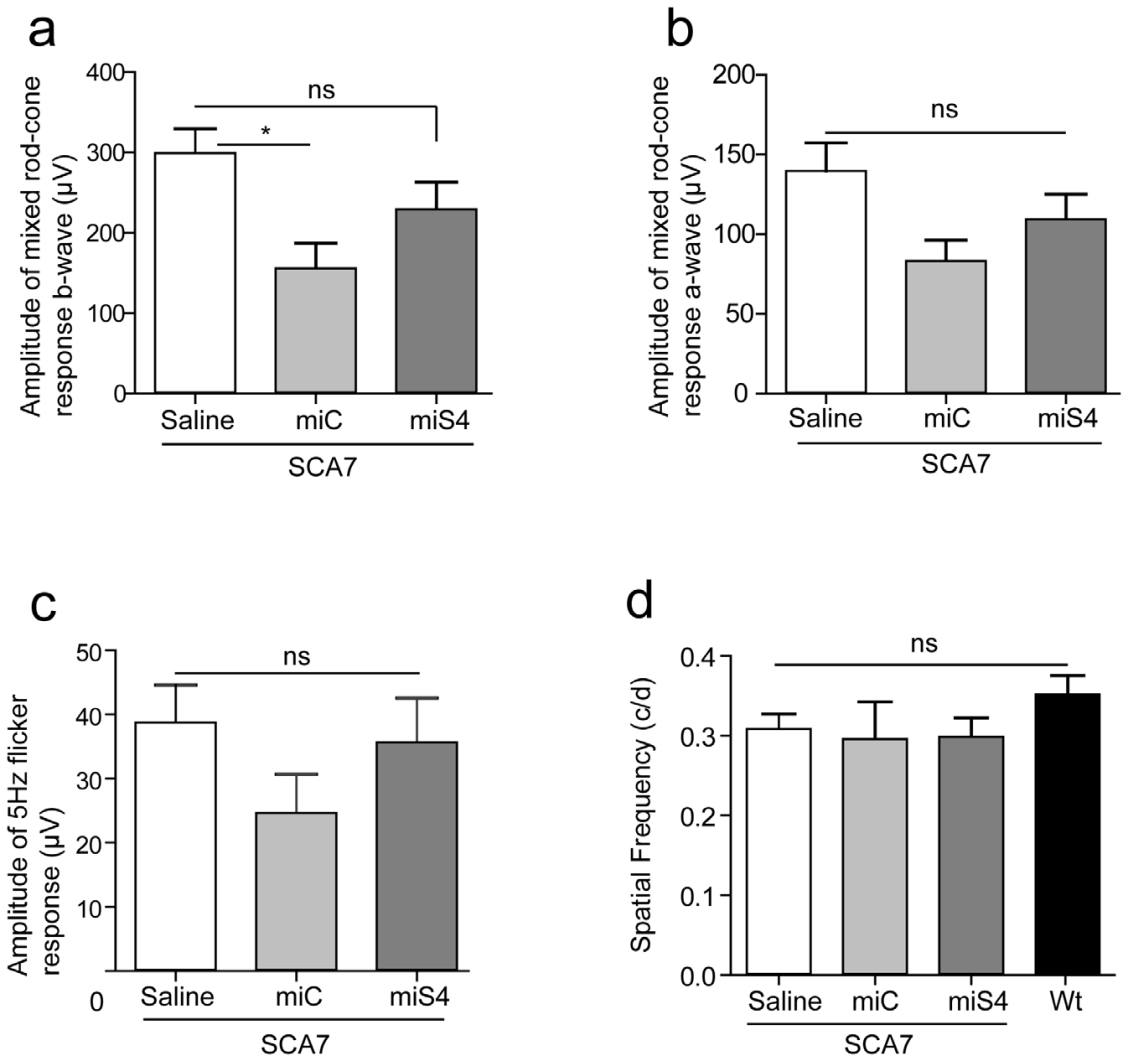
Retinal function 30 weeks post-injection. (a) ERG b-wave of the mixed rod-cone response, n≥4 (b) ERG a-wave of the mixed rod-cone response, n≥4 (c) 5 Hz flicker (cone) response, n≥4 (d) Optokinetic response measured by the spatial frequency, n≥3. Results are represented as mean ±SEM, *p<0.05.

At 30 weeks of age we tested retinal and visual function in SCA7 mice to determine the physiological effects of reducing wildtype and mutant ataxin-7 levels, and the general impact of RNAi on retinal function. This was assessed by electroretinogram (ERG), and optokinetic tracking for visual acuity [10]. Full field ERG recordings showed that the mixed rod-cone response and the isolated cone response were not significantly different in the miS4 injected groups relative to the saline injected controls (Figs. 4a–c). We did observe a significant decline in the mixed rod-cone b-wave response in the miC injected retinas (p<0.05) relative to the saline treated eyes, and hence ERG values were normalized to saline injected controls. There was no difference relative to wildtype mice in the optokinetic tracking response [11] following sustained silencing of ataxin-7 expression (Fig. 4d).

## Discussion

Although the mutant transgene is expressed in the retina in the BAC-Prp-SCA7-92Q mouse, we were surprised to observe no notable retinal phenotype (tested by ERG recordings, funduscopy, histology; this work and data not shown). This may be due to inadequate expression of the transgene in the retinal cells, the background strain of the mice, or alternatively, the mice succumb to cerebellar disease prior to retinal pathology and dysfunction. This is in contrast to the SCA7 phenotype in patients, in which retinal disease precedes the ataxic phenotype in most cases.

Ataxin-7 mRNA is expressed in the outer nuclear layer, inner nuclear layer and ganglion cell layers in the mouse retina and has a similar pattern of expression to the human retina as evidenced by *in situ* hybridization (Fig. S2) [12]. With subretinal injections of AAV2/1, we transduced the outer retina efficiently but not the inner retina. However, since SCA7 is a cone-rod dystrophy, targeting the photoreceptor cells in the outer retina may be sufficient to produce a therapeutic effect. With our therapeutic RNAi sequence targeting mostly the outer retina, as seen by eGFP expression, we achieved significant and sustained reduction of both mutant and wildtype ataxin-7 expression. In Huntington's disease (HD) mice, RNAi suppression of ∼55% mutant and ∼60% wildtype htt mRNA levels in the striatum using artificial miRNAs was sufficient to result in a significant improvement in motor phenotypes and extend lifespan [13]. In SCA1 knockin mice, 58% suppression of mutant *Atxn1* protein levels in the cerebellum was sufficient to rescue motor phenotypes [14]. On the other hand, suppression of ∼34% mutant Atxn3 protein levels in the cerebellum did not improve motor phenotypes but improved molecular phenotypes in SCA3 mice [15]. From these studies of RNAi suppression in polyQ disease models, it would seem that ∼50% suppression of mutant polyQ expression should result in therapeutic benefit at least in targeted tissues of the brain. With the levels of silencing of mutant ataxin-7 (∼50%) in the retina that we observe with miS4, we predict improved retinal function in other SCA7 models with quantifiable retinal disease.

At the outset of this work it was unknown if reducing ataxin-7 expression in the retina would be tolerated. We found that in the miS4 treated SCA7 retinas there was no gliosis and their histological appearance was similar to the normal retina, suggesting that neither the reduction in wildtype ataxin-7 expression levels nor the RNAi sequence induced toxicity. Confirming this, ERG recordings and optokinetic tracking demonstrated that retinal function was not altered in the miS4-injected retinas relative to controls. Therefore, at least this level of ataxin-7 reduction was well tolerated and did not affect retinal structure or function in the BAC-Prp-SCA7-92Q mouse.

Here, we reduced transcript levels by ∼60–70%, which resulted in an ∼45% reduction in the levels of mutant human ataxin-7 protein. In the setting of human SCA7, if treated similarly, we would expect about 50% reduction in both normal and wildtype alleles, leaving 25% normal ataxin-7 protein. Thus, similar studies to those done here should be undertaken in knockin models of SCA7 [16] to fully understand if reducing normal ataxin-7 levels to this degree, in the setting of a mutant allele, is tolerated. An added benefit of testing in this model is that a retinal phenotype is evident, which may allow evaluation of functional rescue. However the knock-in model has a very short lifespan (∼14 weeks), making long term studies impractical. Nonetheless, if reduction of the normal allele to 25% of wildtype levels along with silencing of the mutant allele exacerbates retinal disease, knockdown of both alleles and gene replacement with an RNAi-resistant transgene would be warranted, such as has been done in mice models of autosomal dominant retinitis pigmentosa [17].

The toxic buildup of mutant protein in gain-of-function autosomal dominant diseases such as SCA7 makes them amenable to test gene knockdown therapies. Early gene knockdown strategies using ribozymes, RNA enzymes that cleave specific mRNA molecules, demonstrated delayed photoreceptor degeneration in a rat model of autosomal dominant retinitis pigmentosa [18], [19]. Antisense oligonucleotide and siRNA delivery to the retina can also be used to efficiently silence a transcript and have shown therapeutic benefit [20], [21], [22], [23], [24], however, they are transient and require repeated intraocular delivery. AAV mediated shRNA delivery to the retina, which is a different RNAi trigger to that employed here, has also been shown to be effective [25], [26], however, shRNAs are often toxic in the nervous system and have the potential to saturate the RNAi pathway [27], [28], [29]. On the other hand, AAV mediated delivery of artificial miRNAs or miR-based RNAi sequences have proven to be safe long term [30], [31]. In other work, artificial miRNA sequences were used to target retinal peripherin-2; by 5 weeks post injection, efficient knockdown was observed, however toxicity was not evaluated [32]. Here, we analyzed the long term safety of AAV mediated artificial miRNA expression in the retina 23 weeks post injection and found that our artificial miRNA does not induce toxicity while effectively reducing ataxin-7 levels.

In addition to the KI model of SCA7, the R7E transgenic mouse model generated by Yvert *et al*., has a retinal phenotype [33]. These mice express high levels of mutant ataxin-7 (90Q) exclusively in the rod photoreceptors. These mice show a decline in the ERG a-wave and nuclear inclusions. But despite disorganization of the outer segments, the overall thickness of the ONL is unchanged and there is no abnormal migration of the RPE as seen in SCA7 patients. These mice also differ from human SCA7 patients in that they do not express mutant ataxin-7 in the inner retina.

Currently, there is no restorative or protective therapy for vision loss in SCA7 patients. Therapeutic methods that are being considered in other cone-rod dystrophies and retinal degenerative disease where photoreceptor cells are lost early include stem cell derived photoreceptor cell replacement, electronic retinal implants and optogenetics [34], [35]. RNAi based therapy for SCA7 is a disease modifying therapy that could be applied while photoreceptor cells are still present in the patient, even after dysfunction is noted. Cumulatively our data suggest that non-allele specific silencing of ataxin-7 in the retina by RNAi may be a viable therapeutic strategy in patients with SCA7 retinal degeneration.

## Materials and Methods

### Animals

The University of Iowa Animal Care and Use Committee (IACUC) approved all animal protocols. BAC-PrP-SCA7-92Q transgenic mice were generated in the La Spada lab and were maintained on the C57BL/6J background. Mice were genotyped using primers specific for the mutant human ataxin-7 transgene [6]. Hemizygous and age-matched wild type littermates were used for the experiments. Mice were housed in a controlled temperature environment on a 12-hour light/dark cycle. Food and water were provided *ad libitum*.

### Viral vectors and *in vitro* screening

The plasmids expressing mouse U6-driven artificial miRNA miS4 and miC was cloned as previously described using DNA oligonucleotides [9] using the following primers – S4 forward primer: AAAACTCGAGTGAGCGGGGCTCAGGAAAGAAACGCAAACTGTAAAGCCACAGATGGG, S4 Reverse Primer: AAAAACTAGTAGGCGCGGCTCAGGAAAGAAACGCAAACCCATCTGTGGCTTTACAG Artificial miRNA plasmids were used for *in vitro* screening in HEK293 cells and neuro2a cells. HEK293 cells were co-transfected with myc-tagged human mutant ataxin-7 (92Q) and several artificial miRNA plasmids (C, mm- scrambled controls, S1-S5) or U6 (empty vector control) and untransfected (unt) cells were used as a negative control. 24 hours post transfection, protein was harvested from the whole cell population and ataxin-7 protein levels were analyzed by western blot using a myc antibody. Neuro2a cells were transfected with U6, miC or miS4 artificial miRNA expression plasmids. 24 hours post transfection RNA was harvested from the whole cell population for RT-qPCR analysis. Artificial miRNA expression cassettes were cloned into pAAVmcsCMVeGFP plasmids which co-expressed CMV-driven eGFP [9]. Recombinant AAV serotype 2/1 vectors (AAV.miC.eGFP and AAV.miS4.eGFP) were generated by the University of Iowa Vector Core facility, as previously described [36]. AAV vectors were dialyzed and resuspended in Formulation Buffer 18 (University of Iowa Gene Transfer Vector Core, Iowa City, IA) and titers (viral genomes/ml) were determined by RT-qPCR.

### AAV injections

SCA7 transgenic mice were injected subretinally with 2 µl of AAV1 virus (at 4.26×10^12^ viral genomes/ml) or saline. Briefly, mice were anesthetized using a ketamine/xylazine mix and one drop of 50% betadine solution and topical 1% proparacaine was applied to anesthetize the eye. A sharp 30 gauge needle was inserted through the posterior sclera of the eye followed by a blunt 33 gauge needle inserted into the subretinal space and 2 µL of virus was injected under direct visualization using an operating microscope. A retinal bleb could be seen following successful placement of the injections. Following the injections, a topical antibiotic/steroid ointment was applied and the mice were allowed to recover according to the University of Iowa Animal Care and Use Committee's (IACUC) guidelines for Post-Anesthesia Monitoring, including monitoring of breathing and muscle tone.

### Tissue harvesting and histology

To harvest the retinas, mice were anesthetized with a ketamine/xylazine mix and sacrificed and the eyes removed. The eyes were then fixed in 4% PFA for histological analysis or eGFP expressing retina tissue were dissected under a fluorescence microscope and put in TRIzol (Life Technologies, Grand Island, NY) for RNA isolation. RNA quantity and quality were measured using a NanoDrop ND-1000 (NanoDrop, Wilmington, DE). For histological analysis, the fixed eyes were enucleated and the eye cup was infiltrated in acrylamide embedding solution (1.2 M acrylamide, 0.9 mM bisacrylamide, 0.7% N,N,N',N'-tetramethylethylenediamine, 1 mM MgCl_2_, 1 mM CaCl_2_) overnight and polymerized the next day. The acrylamide was trimmed to the eye cup and placed in OCT (Tissue-Tek). 7 µm thick retinal sections were cut using the Leica Microm Cryostat and used for histological analysis.

Standard hematoxylin and eosin staining was performed on 7 µm sections (n = 3 eyes per group) mounted onto Superfrost Plus slides (Fischer Scientific, Pittsburgh, PA). Images were captured on Leica DMR microscope connected to an Olympus DP72 camera using the Olympus DP2-BSW software (Olympus, Melville, NY).

### *In situ* hybridization

Human donor eyes were obtained through the Iowa Lions eye bank (Iowa City, IA) with informed consent in accordance with the declaration of Helsinki. Tissues were processed as described previously [37] and 7 µm thick retinal sections were cut using the Leica Microm Cryostat. A modified 2′OMe ZEN ataxin-7 probe [38] (Integrated DNA Technologies, Coralville, IA) was used to hybridize to ataxin-7 mRNA in the retina. Retinal sections from three C57Bl/6J mice and human retina were used for *in situ* hybridization using previously described methods [39]. Scrambled probes (mUmGmU mAmAmC mAmCmG mUmCmU mAmUmA mCmGmC mCmC) were used as controls to the ataxin-7 probe (mCmCmUmCmCmUmCmAmCmUmGmGmAmUmAmAmCmCmGmAmGmAmAmGmCmUmGmGmCmUmCmAmGmUmG).

### PCR and western blotting

First-strand cDNA synthesis was performed using total RNA (High Capacity cDNA Reverse Transcription Kit; Life Technologies, Grand Island, NY) as per manufacturer's instructions. RT-qPCR assays were performed on a sequence detection system using primers/probe sets specific for human or mouse ataxin-7, mouse GFAP or mouse β-actin (ABI Prism 7900 HT, TaqMan 2Xuniversal master mix and power SYBR green PCR master mix, Life Technologies, Grand Island, NY). RT-qPCR values were normalized to mouse β-actin. Stem loop PCR was performed as describer earlier [40]. Briefly, PCR primers were designed to identify miS4. Reverse transcription was performed with RT specific primers (S4:GTCGTATCCAGTGCAGGGTCCGAGGTATTCGCACTGGATACGACGGCTCA) using the High Capacity cDNA Reverse Transcription Kit; Life Technologies, Grand Island, NY and cDNA obtained was subject to PCR using specific forward primers (S4 Fwd: GCCCTTTGCGTTTCTTTCC) and a reverse primer (5′ GTGCAGGGTCCGAGGT).

Protein was harvested from whole retinas injected with either AAV2/1 miC or miS4 one month post-injection. Western blots were performed using the polyclonal rabbit anti-ATXN7 (1∶1000; Thermo Fisher Scientific #PA1-749) and β actin (1∶10000, Sigma, A5441) and quantification was performed using Image J software.

### Retinal assays

#### Electroretinogram (ERG)

Full-field ERG was performed using the Espion V5 Diagnosys system (Diagnosys LLC, Lowell, MA). Mice were dark adapted and were anesthetized with ketamine/xylazine mix. ERGs were recorded simultaneously from the corneal surface of each eye after pupil dilation (with 1% tropicamide), using gold ring electrodes (Diagnosys) referenced to a needle electrode (Roland Consult, Brandenburg an der Havel, Germany; LKC Technologies Inc., Gaithersburg, MD) placed on the back of the head. Another needle electrode placed in the tail served as the ground. A drop of 2.5% methylcellulose was placed on the corneal surface to ensure electrical contact and to maintain corneal integrity. Body temperature was maintained at a constant temperature of 38°C using a regulated heating pad. All stimuli were presented in a ColorDome (Diagnosys) ganzfeld bowl, and a camera monitored mouse head and electrode positions. Dim red light was used for room illumination until dark-adapted testing was completed. A modified International Society for Clinical Electrophysiology of Vision (ISCEV) protocol [41], [42] was used, including a dark-adapted dim flash of 0.01 cd.s/m^2^, maximal combined response (standard combined response or SCR) to bright flash of 3 cd.s/m^2^, light-adapted bright flash of 3 cd.s/m^2^, and 5-Hz flicker stimuli at 3 cd.s/m^2^. The a-wave was measured from the baseline to the trough of the first negative wave. The b-wave was measured from the trough of the a-wave to the peak of the first positive wave, or from the baseline to the peak of the first positive wave if no a-wave was present.

#### Optokinetic tracking

The optokinetic response was measured using an OptoMotry system according to previously described methods (Cerebral Mechanics, Lethbridge, Alberta, Canada) [11]. Briefly, mice were placed onto an elevated platform in the test chamber, and tested after ∼60 seconds of brief acclimation. Mice were presented with a vertically oriented sine wave grating rotating at 12°/s. The between stimulus ‘blank’ was an equal luminance gray homogenous surround (152 cd.sm^2^). The level of contrast was fixed at 100%, and the spatial frequency was varied to determine thresholds for stimulus detection using a standard protocol. Spatial frequency was expressed in cycles per degree (c/d) for one sine wave (paired vertical black and white bars). Tracking head movements were scored by an experienced operator blind to genotype. Test sessions were during the mid 4 hours of the light phase of a daily cycle. The test chamber was thoroughly cleaned between animals. Where no response was observed, an animal was tested at least 10 times.

#### Optical coherence tomography (OCT)

(Bioptigen, Research Triangle Park, NC) was performed after placing the animals under ketamine/xylazine anesthesia as described above. 1% tropicamide was used to dilate pupils, and the retinas were scanned. Methylcellulose lubricant was placed on the corneas, and the noncontact probe was positioned near the cornea until the retinal image could be seen on the screen. This was then focused and oriented with the optic nerve (ON) in the middle of the scan as a landmark. Retinal OCT was performed using rectangular volume scan (volumetric acquisition made up of a series of B-scans) with a length of 1.40 mm at a width of 1.40 mm at a rate of 1000 A-scan/B-scan. An average of four repeated B scans (of the same region) centered on the ON was used for analysis. The total retinal thickness was measured on the OCT image at two locations, each 0.3 mm from the edge of the ON, on either side using the measuring tool provided (Bioptigen). Eyes were excluded from further analysis if retinal disruption more than four times the area of a typical injection site due to hemorrhage or chronic retinal detachment (greater than 1 week after injection) was noted on OCT.

### Figure preparation and statistical analysis

All photographs were formatted with Adobe Photoshop software, all graphs were made with Graphpad Prism software, and all figures were constructed with Adobe Illustrator software. All assays were analyzed by one-way ANOVA with Bonferroni post-hoc tests, with exception of the western blotting quantification that was analyzed by the Students t-test.

## Supporting Information

Figure S1 (a) HEK293 cells co-transfected with myc tagged human mutant ataxin-7 and several artificial miRNA plasmids (C, mm- scrambled controls, S1–S5) or U6 (empty vector control) and untransfected (unt) cells were used as a negative control. 24 hours post transfection, protein was harvested and ataxin-7 protein levels were analyzed by western blot using a myc antibody (n = 3). A representative western blot is shown. (b) Neuro2a cells were transfected with U6, miC or miS4 expression constructs. 24 hours post transfection RNA was harvested for RT-qPCR analysis. Results are represented as mean ±SEM (n = 3), *p<0.05.
(PDF)

Figure S2 **Expression of ataxin-7 mRNA in mouse and human retinas.** *In situ* mRNA analysis for detection of ataxin-7 mRNA expression in mouse (n = 3) and human retina (n = 3). Scrambled probes were used as controls.
(PDF)
