# Supplementary figures and images for: RNA Interference-Based Therapy for Spinocerebellar Ataxia Type 7 Retinal Degeneration
Source: PLoS One. 2014 Apr 23;9(4):e95362. doi: 10.1371/journal.pone.0095362 (PMC3997397; doi:10.1371/journal.pone.0095362)

**a**

U6 C S1 S2 S3 S4 HS5 mm unt

myc

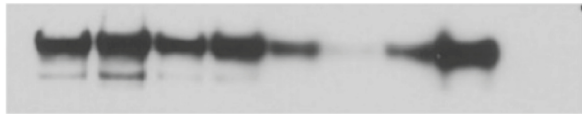 $\beta$ -actin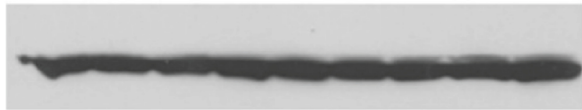**b**

Relative mouse ataxin-7 mRNA levels

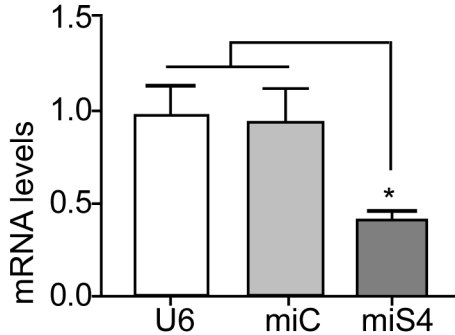

Supplement: Figure S1 — (a) HEK293 cells co-transfected with myc tagged human mutant ataxin-7 and several artificial miRNA plasmids (C, mm- scrambled controls, S1–S5) or U6 (empty vector control) and untransfected (unt) cells were used as a negative control. 24 hours post transfection, protein was harvested and ataxin-7 protein levels were analyzed by western blot using a myc antibody (n = 3). A representative western blot is shown. (b) Neuro2a cells were transfected with U6, miC or miS4 expression constructs. 24 hours post transfection RNA was harvested for RT-qPCR analysis. Results are represented as mean ±SEM (n = 3), *p<0.05. (PDF) [file pone.0095362.s001.pdf]

## Mouse

control

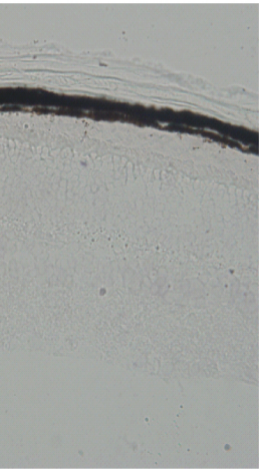

ataxin-7

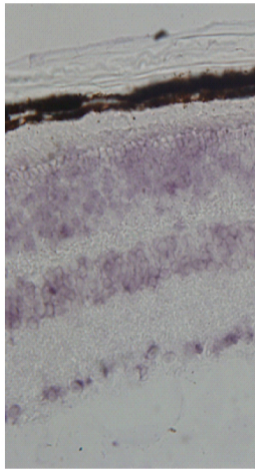

RPE

ONL

INL

GCL

## Human

control

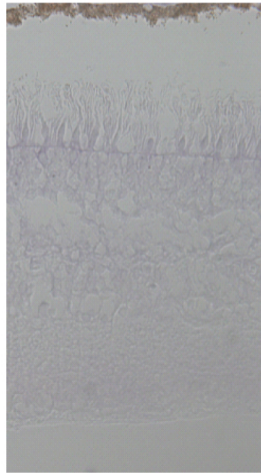

ataxin-7

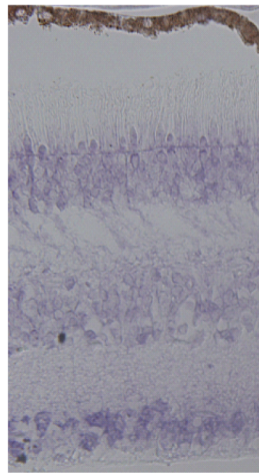

RPE

ONL

INL

GCL

Supplement: Figure S2 — Expression of ataxin-7 mRNA in mouse and human retinas. In situ mRNA analysis for detection of ataxin-7 mRNA expression in mouse (n = 3) and human retina (n = 3). Scrambled probes were used as controls. (PDF) [file pone.0095362.s002.pdf]
